# Norethisterone‐induced cholestasis: A case report

**DOI:** 10.1002/ccr3.5522

**Published:** 2022-03-03

**Authors:** Safa Moussaoui, Mehdi Abdelwahed, Nabil Ben Chaaben, Ahlem Bellalah, Najah Ben Fadhel, Arwa Guediche, Mejda Zakhama, Ramzi Tababi, Karim Aouam, Zakhama Abdelfattah, Hichem Loghmari, Leila Safer

**Affiliations:** ^1^ 314309 Department of Gastroenterology Fattouma Bourguiba University Hospital of Monastir Tunisia; ^2^ 314309 Department of Pathology Fattouma Bourguiba University Hospital of Monastir Tunisia; ^3^ Department of Pharmacology Monastir‐Faculty of medicine Tunisia

**Keywords:** cholestasis, contraceptive pills, norethisterone, progesteron

## Abstract

Case presentation

This case report concerns a 49‐year‐old woman who developed cholestasis (build‐up of bile in the liver) two months and a half after initiating norethisterone, progestin‐only pills, which resolved after the withdrawal of these pills.

## Graphical abstract

The estrogenic component of combined oral contraceptive pill is the one generally believed to be responsible for drug‐induced cholestasis. Progesteron‐only induced‐cholestasis is quite rare and could be triggered by underlying predispositions.

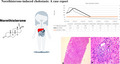

## 1 INTRODUCTION

Thousands of drugs have been reported to cause different varieties of liver disorders.^1^ Establishing their causality has been the major hindrance in understanding the mechanisms of drug‐induced‐liver injury. In fact, the cholestatic injury is one of the most severe manifestations of drug‐induced liver disease (DILD), which may occur particularly under some conditions of genetic alterations in the expression of enzymes or transporters.^2^

A wide variety of commonly used drugs are known to induce such side effects. Nevertheless, a combination of oral contraceptive steroids is rarely associated with cholestasis, which resembles intrahepatic cholestasis of pregnancy. In fact, the estrogenic component of the combined oral contraceptive pill is believed to be responsible. On the contrary, high doses of progestin can lead to liver enzyme elevation, which usually occurs 1 to 2 weeks after initiating the treatment which consists mostly of serum aminotransferase elevation with no changes in the alkaline phosphatase or bilirubin. We report a case of cholestasis induced by norethisterone, a progestin contraceptive, which resolved after the withdrawal of these pills.

## 2 CASE REPORT

A 49‐year‐old women was admitted to our gastroenterology unit in December 2020 for jaundice with pruritus. The medical history showed a hepatic hydatid cyst operated 20 years ago neither a family health history of liver disease nor a personal history of alcohol use or substance abuse. Our patient had a metrorrhagia for which she was prescribed Primolut—Nor a progestin—only contraceptive initiated on the first of October 2020. Two and a half months later, she presented with jaundice, tiredness, and intense pruritus. The symptoms, which appeared 10 days before she was admitted, included dark urine but she had neither abdominal pain nor fever. A physical examination showed slight jaundice associated with scratching lesions. Then, an abdominal examination showed no hepatosplenomegaly.

The results revealed by the blood tests are shown in Table 1.

**TABLE 1:** Blood test results

Laboratory parameters	Result unit	Reference range
Hemoglobin	11,5 g/dL	12–14
Eosinophilia	200 cells/mcL	<500
total bilirubin	127 mmol/L	<17
direct bilirubin	89 mmol/L	<5
alkaline phosphatase	240 U/L	50–150
Gamma‐glutamyl transferase(GGt)	30 U/L	7–64
prothrombin time	90%	>70%
aspartate aminotransferase	23 U/L	5–45
alanine aminotransferase	30 U/L	5–45

**FIGURE 1:**
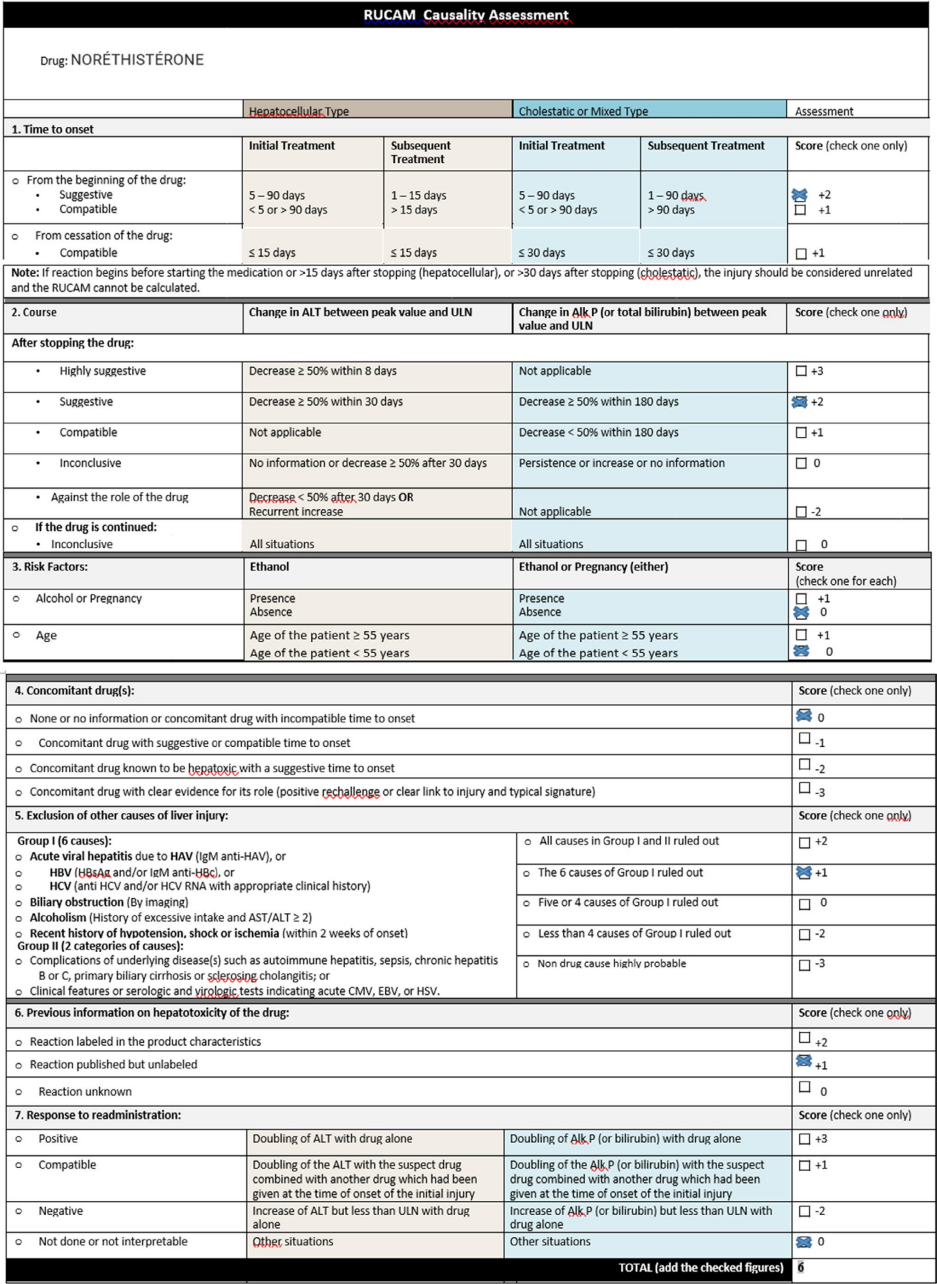
Roussel Uclaf Causality Assessment Method (RUCAM) according to our case report Abbreviations: ALT, Alanine Aminotransferase; Alk P, Alkaline Phosphatase; ULN, Upper Limit of the Normal range of values.

**FIGURE 2:**
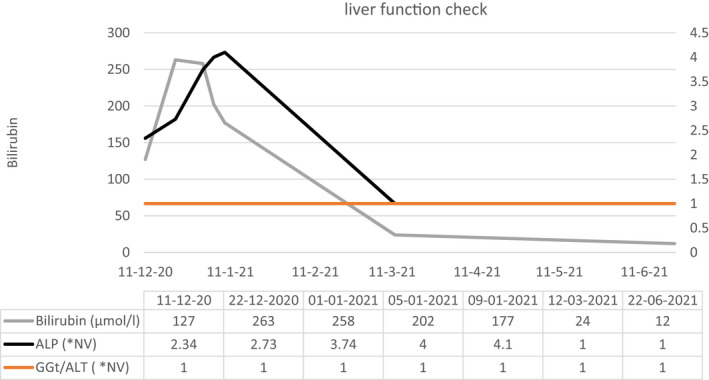
Hepatic test during disease monitoring Abbreviations: ALP, Alkaline Phosphatase, ALT: alanine aminotransferase, GGT:Gamma‐glutamyl transferase.

Abdominal ultrasound imaging showed no abnormalities, with no signs of bile duct dilatation. However, an etiological assessment of intrahepatic cholestasis revealed:

- negative serologic tests for acute and chronic viral hepatitis (B,C,A, and E),
- negative anti‐nuclear antibody, anti‐mitochondrial antibody (AMA), and anti‐smooth muscle antibody.
- Magnetic resonance imaging (MRI) of the biliary tract was normal then, a

full investigation revealed no cause for the woman's jaundice except for contraceptive pill and with RUCAM score = 6 (Figure 1), which showed that the contraceptive pills were deemed to be responsible. As a consequence, the woman was asked to stop taking norethisterone pill and instead, she received a symptomatic treatment to relieve pruritus based on antihistamines and empirical course of rifampicin 400 mg daily because it inhibits the uptake of bile acids by hepatocytes, which led to a rapid clinical improvement and a slower biochemical response (Figure 2).

**FIGURE 3:**
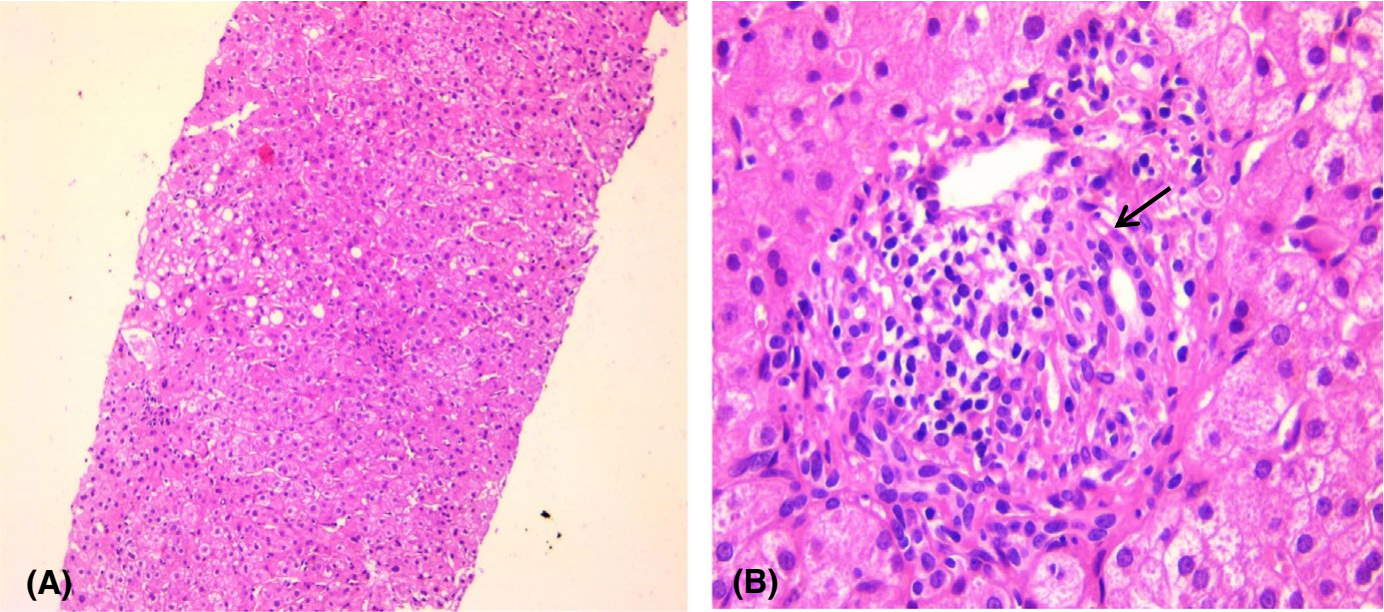
Histology results showed A/ Foci of steatosis and intralobular lymphocytic infiltrate (HE × 100). B/ Portal inflammation with lymphocytic cholangitis (Arrow) (HE × 400)

Liver biopsy showed portal and intralobular inflammation with images of lymphocytic cholangitis and slight macrovacuolar steatosis.(Figure 3).

In fact, six months later, the woman's symptoms subsided completely, and her liver biochemistry was normal.

## 3 DISCUSSION

In this section, we present a case of cholestatic type Drug Induced Liver Injury (DILI) due to norethisterone. In fact, this diagnosis was retained for several reasons; First of all, the relatively long period of pruritus antedating the onset of jaundice, which occurred two and a half months after the initiation of the treatment, was in conformity with cases published in literature.^3^, ^4^ Secondly, a progressive scanning was used after the cessation of taking progesterone pills. Our patient also presented with normal GGt level with histological results consisting in the presence of portal and intralobular inflammation with images of lymphocytic cholangitis.

The normality of GGT level in intrahepatic cholestasis is exceptional and tends to occur in infants or young children with some types of familial intrahepatic cholestasis, such as progressive familial intrahepatic cholestasis types 1 and 2 or in adenosine triphosphate‐binding cassette (ABCB4) mutations. Benign recurrent intrahepatic cholestasis can also lead to progressive familial intrahepatic cholestasis with low serum GGT.^5^, ^6^

Oral contraceptives are currently available under a combination of both an estrogen and a progestin or progestin‐only preparations on the market. The estrogen component is either ethinyl‐estradiol or mestranol. The progestin component is one of six commercially available 19‐nortestosterone derivatives (norethindrone, ethynodiol diacetate, norethynodrel, norgestrel, Ievonorgestrel, or norethindrone acetate).^7^

Liver‐exposure to high levels of sex steroids via the portal circulation puts this organ at risk for the adverse effects of these drugs. The majority of women contraceptives taking oral would not have hepatobiliary effects while only a small number of them may sustain hepatic changes varying from benign intrahepatic cholestasis to the development of benign or even malignant tumors of the liver while oral contraceptive acute cholestatic injury has become less common as the doses of estrogens and progestin have been reduced in more recent formulations. In fact, the estrogen component of combined oral contraceptive pills is usually described as responsible for the rare development of intrahepatic cholestasis.^8^

Moreover, estrogen induced cholestasis was detected especially in women with a history of idiopathic cholestasis of pregnancy and a potential genetic component involving the bile salt export pump (BSEP) and ATP‐binding cassette sub‐family B 11 member gene (ABC B11).^9^, ^10^ Studies in rats suggest that canalicular bile transporters, such as multidrug resistant protein 2 (MRP‐2), which is responsible for biliary secretion of bilirubin glucuronides, may be involved in the estrogen‐induced cholestasis.^11^

Unlike estrogens, progestogens are rarely involved in cholestasis.^3^, ^4^ In fact, it is generally assumed that progesterone‐only contraceptives are safe therefore, it would be a solution to avoid recurrence of hepatic problem previously described with combined contraception. However, there are reports that intrahepatic cholestasis with high doses of progesterones is used to treat women with breast cancer.^12^ Furthermore, the serum concentration of sulfated metabolites of progesterone is known to be elevated in patients with obstetric cholestasis and large amounts of sulfated progesterone metabolites excreted in their urine, which suggested that progesterone metabolites may have an important role than estradiol metabolites in the pathogenesis of obstetric cholestasis.^13^, ^14^

Moreover, the mechanism of cholestasis induced by progestogens could be explained by a reduction of Na+‐dependent and Na+‐independent influx of the primary bile acid taurocholate into primary human.^10^, ^15^ On the contrary, drug‐induced cholestasis can have several histologic features with the most common form is cholestatic hepatitis. Then, its second form is bland cholestasis, typically associated with oral contraceptives and anabolic steroids, which is characterized by canalicular dilatation and bile plugs but without significant inflammation.^16^

However, oral contraceptive acute cholestatic injury has become less common as the doses of estrogen and progestin have been reduced in more recent formulations. In fact, this steroid can trigger a drug‐induced cholestasis in a susceptible patient presenting a cholangiopathy due to steatosis and /or genetic predispositions.

Unfortunately, one of the limitations of this case report was the inability to study genetics and predisposing factors for progesterone drug‐induced cholestasis in this patient.

## 4 CONCLUSION

Progesteron‐only contraceptive pills are frequently prescribed for hormone imbalance and reversible means of birth control. Nevertheless, unlike estrogen, progesterone induced‐drug cholestasis is quite a rare complication, which could be triggered by underlying predispositions.

## CONFLICT OF INTEREST

The authors declare that they have no competing interests.

## AUTHOR CONTRIBUTIONS

SM,N.BC, MA,LS, MZ, and AG were responsible of the patient follow‐up and writing the manuscript. AZ and AB carried out the anatomic pathology studies. KA and N.BF carried out pharmacovigilance investigation. RT took care of the patient during her hospitalization. All authors read and approved the final manuscript.

## ETHICAL APPROVAL

Ethics approval and consent to participate. This article was approved by the ethics committee of Fattouma Bourguiba Hospital.

## CONSENT

Written informed consent was obtained from the patient for the publication of this case report and any accompanying images. A copy of the written consent is available for review by the Editor‐in‐Chief of this journal.

## Data Availability

Supporting data are available in Gastroenterology department in Fattouma Bourguiba Hospital, Monastir,Tunisia.
